# c-Myb Dominates TBK1-Mediated Endotoxin Tolerance in Kupffer Cells by Negatively Regulating DTX4

**DOI:** 10.1155/2023/5990156

**Published:** 2023-03-31

**Authors:** Yi-Lin Wu, Le-Han Pan, Zhu-Jun Yi, Wen-Feng Zhang, Jian-Ping Gong

**Affiliations:** ^1^Department of Hepatobiliary Surgery, Second Affiliated Hospital, Chongqing Medical University, Chongqing 400016, China; ^2^Department of Hepatobiliary Surgery, Chongqing University Three Gorges Hospital, Chongqing 404100, China

## Abstract

As a protective mechanism regulating excessive inflammation, endotoxin tolerance plays a vital role in regulating endotoxin shock. Kupffer cells are players in mediating endotoxin tolerance. Nonetheless, the regulatory mechanism regulating endotoxin tolerance is barely known. A nonclassical IKK kinase called TRAF-associated NF-*κ*B activator (TANK)-binding kinase 1 (TBK1) can regulate inflammation. Here, we found that TBK1 is required for endotoxin tolerance in Kupffer cells. TBK1 plays a dominant role in regulating endotoxin tolerance by negatively regulating the induction of p100 processing. Deltex E3 ubiquitin ligase 4 (DTX4), a negative regulator of TBK1, can promote TBK1 K48-mediated ubiquitination and indirectly regulate endotoxin tolerance in Kupffer cells. We demonstrate that the c-Myb transcription factor could negatively regulate DTX4. Overexpression of c-Myb can be used to reduce the ubiquitination of TBK1 by reducing DTX4 transcription and to boost the anti-inflammatory effect of endotoxin tolerance. Thus, this study reveals a novel theory of TBK1-mediated endotoxin tolerance in Kupffer cells.

## 1. Introduction

Inflammation is an intricate pathophysiological state based on immune cells responding to infection or tissue damage. However, excessive inflammation can lead to severe pathological shapes, for example, autoimmune diseases, metabolic diseases, and sepsis. The mechanism involved in gaining control of excessive inflammation is prominent and is required by the host to resist endotoxic shock. The most well-studied and most important defense mechanism to date is endotoxin tolerance (ET) [1, 2]. Cells and organisms are stimulated by exposure to low concentrations of endotoxin (for example, lipopolysaccharide (LPS)). Cells and organisms exhibit a temporary immune no-/low-response phenomenon, which is called ET. ET is strongly associated with nuclear factor-*κ*B (NF-*κ*B) plasticity at different stages of the inflammatory response [3, 4]. The plasticity of NF-*κ*B may drive monocytes/macrophages to develop different phenotypes and transition from an inflammatory state to an anti-inflammatory (perhaps endotoxin-resistant) condition [1, 5]. Kupffer cells (KCs) serve as the main immune cells and play a prominent role in liver inflammation [6].

TRAF-associated NF-*κ*B activator-binding kinase 1 (TBK1) is an IKK-related serine/threonine kinase that is ubiquitously present in all tissues. In contrast to classical IKK kinases (IKK*α*, IKK*β*), TBK1 and IKK*ε*, nonclassical IKK kinases, are activated by a MyD88-independent pathway [7]. The MyD88-independent pathway can activate TBK1 by recruiting TRAF proteins. Activation of TBK1 and IKK*ɛ* phosphorylates interferon regulatory factor 3 (IRF3) and leads to the expression of IFN-*α* and IFN-*β*. Many innate immune signaling pathways can activate TBK1, such as TLR4-TRIF by LPS, RIG-I-MAVS by viral RNA, cGAS-STING by double-stranded DNA (dsDNA), and TLR3-TRIF by dsRNA [8]. TBK1 is a noncanonical IKK that regulates inflammation. TBK1 can induce the degradation of I*κ*B and NF-*κ*B activation through phosphorylating IKK*β*. TBK1 overexpression induces nuclear translocation of NF-*κ*B RelA subunit (p65). After TBK1 short hairpin RNA (shRNA) knockdown, the Ser536 NF-*κ*B phosphorylation level can be significantly reduced by about 50% [9, 10]. However, it was also shown that TBK1 is not essential for NF-*κ*B activation because TBK1 phosphorylates only one of the two regulatory serines required for I*κ*B degradation [7]. TBK1 appears to play a contradictory role in NF-*κ*B regulation. Mice lacking TBK1 die from extensive hepatocyte apoptosis during embryonic development, making it difficult to determine the role of TBK1 [11]. We hypothesize that TBK1 function is highly dependent on the cellular and signaling context. Therefore, the role of TBK1 in endotoxin resistance deserves attention and confirmation.

To precisely regulate TBK1 in the inflammatory signaling pathway, the activity of TBK1 is accurately modulated by multiple posttranslational modifications. The most common form of regulation is to modulate TBK1 activity by altering the phosphorylation state of TBK1, such as SHIP1, PPM1B, and glucocorticoids [12]. Apart from phosphorylation, TBK1 can be regulated by ubiquitination. Other E3 ubiquitin ligases, such as TRAF3 or Mind bomb (MIB), play key roles in K63-linked TBK1 ubiquitination, promoting TBK1 activation shortly after infection [13, 14]. Subsequently, NLRP4/deltex E3 ubiquitin ligase 4 (DTX4) and another TRAF-interacting E3 protein ligase (TRIP) induce polyubiquitination of K48-linked TBK1, leading to its degradation in the proteasome. [15, 16]. It is reasonable to believe that DTX4 is one of the most paramount negative regulators of the ubiquitination/degradation of TBK1.

TBK1 can be quickly regulated by regulating DTX4 at the transcription level. Using the DTX4 transcription factor prediction database, we identified the c-Myb transcription factor, a nuclear transcription factor associated with cell proliferation and differentiation. Here, we demonstrate that c-Myb can regulate TBK1-mediated ET in KCs by negatively regulating DTX4. ET requires TBK1 phosphorylation, and TBK1 deficiency in KCs prevents ET, resulting in increased production of proinflammatory cytokines.

## 2. Materials and Methods

### 2.1. Reagents

Anti-c-Myb, DTX4, TBK1, Phospho-TBK1/NAK (Ser172), inducible nitric oxide synthase (iNOS), tumor necrosis factor-*α* (TNF-*α*), and ubiquitin (linkage-specific K48) antibodies were obtained from Abcam (Cambridge, MA, UK). Antibodies against p-TBK1/NAK (Ser172), p-NF-*κ*B p65 (Ser536), NIK, NF-*κ*B2 p100/p52, p-NF-*κ*B2 p100 (Ser866/870), IKK*α*, IKK*β*, p-IKK*α*/*β* (Ser176/180), and the DYKDDDDK tag were purchased from CST (Danvers, MA, USA). Anti-NF-*κ*B, GAPDH, and *β*-actin antibodies were obtained from Servicebio (Wuhan, China). Anti-NLRP4 (catalog #NB100-56156) was purchased from Novus Biologicals (USA).  Table 1 shows the information on the relevant antibodies. Secondary antibodies were provided by Boster Biological Technology (Wuhan, China). The TNF-*α* mouse-uncoated enzyme-linked immunosorbent assay (ELISA) kit was purchased from Thermo Fisher Scientific. The mouse interleukin-10 (IL-10) ELISA kit was produced by Lianke Sciences (Hangzhou, China). The mouse IL-6, IFN-*α*, and IFN-*β* ELISA kits were produced by Ruixin Biotechnology (Quanzhou, China). Glutamate oxaloacetate transaminase (GOT) and glutamate pyruvate transaminase (GPT) assay kit (BC1565, BC1555) were purchased from Solarbio (Beijing, China). MRT67307 was purchased from MCE (MedChemExpress, Shanghai, China). LPS (L2880) was provided by Sigma-Aldrich (St. Louis, MO, USA). Liposomes (F70101C-NC-2) were obtained from FormuMax. TBK1 small interfering RNA (siRNA) (m) was purchased from Lab Cell (Chongqing, China). c-Myb shRNA (m) lentiviral particles were purchased from GeneChem (Shanghai, China).

### 2.2. Animals and Protocols

All animal experiments followed the National Institutes of Health guidelines. Male C57BL/6 mice (8–10 weeks old, 22 ± 2 g) were randomly assigned to the following groups of 15 mice each [17]:

1. Normal saline (NS) group: Intraperitoneal saline injection as control.
2. ET group: 50, 250, and 500 *µ*g/kg LPS were injected intraperitoneally for 3 consecutive days. Twelve hours after the last injection, the dose of LPS becomes 20 mg/kg.
3. Nonendotoxin tolerance (NET) group: Direct use of 20 mg/kg LPS.
4. Dimethyl sulfoxide (DMSO) group: Mice were treated with an intraperitoneal injection of DMSO.
5. MRT67307 + ET (MRT67307 + ET) group: MRT67307 (20 mg/kg) was used before the LPS injection. The rest of the steps are the same as the ET group.

Every 24 hr survival and body weight were collected for 1 week.

### 2.3. Cells and Isolation of KCs

RAW264.7 and HEK293T cells were stored in a complete optimal medium (Dulbecco's Modified Eagle Medium (DMEM) + 10% fetal bovine serum). Cells were divided as follows: the control group (phosphate-buffered saline (PBS)), the ET group (10 ng/mL LPS treated for 24 hr, 1,000 ng/mL LPS treated for 12 hr), and the NET group (only 1,000 ng/mL LPS treated for 12 hr) [17].

KCs were isolated according to a suggested three-step method, including type IV collagenase digestion, gradient centrifugation, and selective adhesion [18]. Cells were grown in six-well plates using DMEM. KCs were treated as mentioned above to establish three groups.

### 2.4. Small Interfering RNA

RNA oligonucleotides were used to synthesize siRNA (Lab Cell, Shanghai, China) with the following sequences: TBK1-siRNA: 5′-GAACGCAGACUAGCUUAUA-3′ and control siRNA: 5′-UAUAAGCUAGUCUGCGUUC-3′. RAW264.7 cells were incubated in the complete optimal medium for 12 hr. The medium was removed from the wells and was replaced with the complete medium (including siRNA and Lipo8000) replacement. Cells required continuous medium exchange to maintain viability. Western blotting (WB) and quantitative polymerase chain reaction (qPCR) were used to determine the effectiveness of siRNA. The ET model was established as previously described.

### 2.5. shRNA Lentiviral Particle Transduction in RAW264.7 Cells

RAW264.7 cells were subjected to c-Myb shRNA (m)-mediated lentiviral transduction according to the GeneChem protocol. The multiplicity of infection (MOI = 20) and dose of puromycin (2.5 *μ*g/kg) were determined by a pre-experiment. Briefly, cells were grown overnight in a complete optimal medium. The medium was removed from the wells and was replaced with the complete optimal medium containing NC vector (GV492: Ubi-MCS-3FLAG-CBh-gcGFP-IRES-puromycin), LV-c-Myb, or LV-c-Myb-RNAi for 12 hr exchange. Cells required continuous medium exchange to maintain viability. Puromycin was used to select stable strains.

### 2.6. Western Blotting Analysis

After lysing, the supernatant obtained by centrifugation (15 min, 14,000 × *g*, 4°C) was mixed with sodium dodecyl sulfate (SDS)–polyacrylamide gel electrophoresis (PAGE) loading buffer and heated. Proteins were separated by SDS–PAGE and electroblotted onto polyvinylidene fluoride membranes. Five percent bovine serum albumin (BSA) was used to block the membranes (1 hr, 37°C). Primary antibodies were incubated overnight at 4°C with the used membranes. The membranes were incubated with a secondary antibody at 37°C for 1 hr after being washed. In the end, the Quantity One gel scanning system was used to detect the proteins.

### 2.7. Reverse Transcription-Polymerase Chain Reaction

Cells were lysed using TRIzol Reagent. After isolation, PrimeScript® RT Reagent Kit with gDNA Eraser (Takara, Japan) was used to perform cDNA synthesis. The reactions were carried out on a Bio-Rad CFX Connect^TM^ Real-Time System. The primer was given as follows: mouse c-Myb, forward 5′-TCCTCCTTCTCCTCCTCCTCCTC-3′, reverse 5′-CAGTCGTCTGTTCCGTTCTGTTCC-3′; mouse DTX4, forward 5′-GCGTCAAGGCTGCTGTGGTC-3′, reverse 5′-CCTTCCAGTCTTCCTGTTGCTTCC-3′; mouse TBK1, forward 5′-TCAGGAAATTTGCCTATTGAAAATTT-3′, reverse 5′-GCTTTGTCTTTCTTGTTATCTTTTAAGTTGT-3′; mouse TNF-*α*, forward 5′-TCAGGAAATTTGCCTATTGAAAATTT-3′, reverse 5′-GCTTTGTCTTTCTTGTTATCTTTTAAGTTGT-3′; mouse IL-6, forward 5′-TACCACTTCACAAGTCGGAGGC-3′, reverse 5′-CTGCAAGTGCATCATCGTTGTTC-3′; *β*-actin: forward 5′-CATTGTGATGGACTCCGGAG-3′; reverse 5′-CTGCCGGTCCAGTAGTATA-3′.

### 2.8. Enzyme-Linked Immunosorbent Assay

TNF-*α*, IL-10, IFN-*α*, and IFN-*β* in the supernatant of cells and TNF-*α* and IFN-*β* in the mouse serum were quantitatively measured using mouse ELISA kits in line with the protocol.

### 2.9. Immunofluorescence Staining

After fixed, cells were permeabilized with 0.3% Triton X-100 for 10 min. Five percent BSA was blocked, and then washed with PBS. Cells were then incubated overnight at 4°C with the primary antibodies. Then, incubated with the secondary antibody (1 : 500) for 1 hr in the dark, and finally incubated with DAPI for 10 min. After blocking the stained cells with a fluorescent quencher, they were visualized with a confocal laser scanning microscope.

### 2.10. Histological Analysis

After being fixed, dehydrated, and embedded, liver tissues were cut. Next, sections were stained with hematoxylin and eosin (H&E). Immunohistochemical staining was performed using a commercial kit. After tissue sections were deparaffinized, hydrated, and antigen retrieved, they were incubated with antibodies overnight at 4°C. IgG polymers, DAB, and hematoxylin were used. After dehydration sealing, tissues were observed with a microscope and images were collected.

### 2.11. Liver Function Analysis

The serum of the different treatment groups was obtained by centrifugation and the aspartate aminotransferase (AST) and alanine aminotransferase (ALT) levels were measured according to commercial kits.

### 2.12. Bioinformatics Analysis

The promoter sequence of DTX4 was obtained from NCBI. The TFBD database (http://bioinfo.life.hust.edu.cn/) and JASPAR website (http://jaspar.genereg.net/sites/MA0093.2/) were used to predict the transcription factors of DTX4. The c-Myb binding sites were predicted from the JASPAR website.

### 2.13. Coimmunoprecipitation

The input material was retained after cell lysis. It took at least 2 hr at 4°C to confirm the binding of the agarose beads to the antibody. The antibody-conjugated agarose beads were added to the cell lysate and were shaked overnight at 4°C. Elution was repeated for nonspecifically bound proteins to obtain sample proteins. The outcome was detected by WB.

### 2.14. Chromatin Immunoprecipitation-qPCR Analysis

The HEK293T cells were cross-linked and quenched. The pellets were lysed after washing with PBS. After centrifugation, the supernatant was discarded, lysed with lysis buffer, and sonicated. Sheared chromatin was incubated overnight with primary antibody bound to the Pierce^TM^ Protein A/G Agarose Beads, followed by elution and reverse cross-linking at 65°C overnight. TE buffer was added to the DNA elution buffer, treated with RNase and proteinase K, and the DNA was isolated and purified subsequently. Since there was no antibody against c-Myb available for ChIP, the c-Myb plasmids fused to the FLAG tag were analyzed by the ChIP-qPCR assay.

### 2.15. Dual-Luciferase Reporting System

HEK293T cells were transfected with the dual-luciferase reporter construct pcDNA3.1 + pGL3-DTX4-WT1 + PRL-TK or the luciferase reporter construct pcDNA3.1-c-Myb + pGL3-DTX4-WT1 + PRL-TK and the internal control vector pRL-TK at a ratio of 20 : 1 (reporter construct:control vector) using Lipofectamine^TM^ 2000. After 5 hr, the medium was changed containing 6 *μ*M of curcumin, including 100% DMSO.

### 2.16. Immunostaining of TUNEL

The number of apoptotic cells in paraffin sections was detected by TUNEL using the TUNEL kit (Beyotime, C1088). The sections were deparaffinized in xylene and eluted with ethyl alcohol. Then, after proteinase K repairing, permeabilized with 0.1% Triton X-100, TDT was used. Finally, the tissue was incubated with DAPI in the dark. Stained cells were observed by fluorescence microscope after sealing.

### 2.17. Statistical Analysis

Prism software (GraphPad Prism version 5.0a) was used to perform the statistical analysis. The average of at least three independent samples was used to calculate the standard error of the mean. Depending on the experimental design, one-way analysis of variance (ANOVA) with Dunnett's multiple comparisons or a two-tailed Student *t*-test was used to test the statistical significance. *p*-Value <0.05 was considered as statistically significant ( ^*∗*^*p* < 0.05,  ^*∗∗*^*p* < 0.01,  ^*∗∗∗*^*p* < 0.001,  ^*∗∗∗∗*^*p* < 0.0001).

## 3. Results

### 3.1. TBK1 Is Required for KCs Endotoxin Tolerance In Vitro

**Figure 1:**
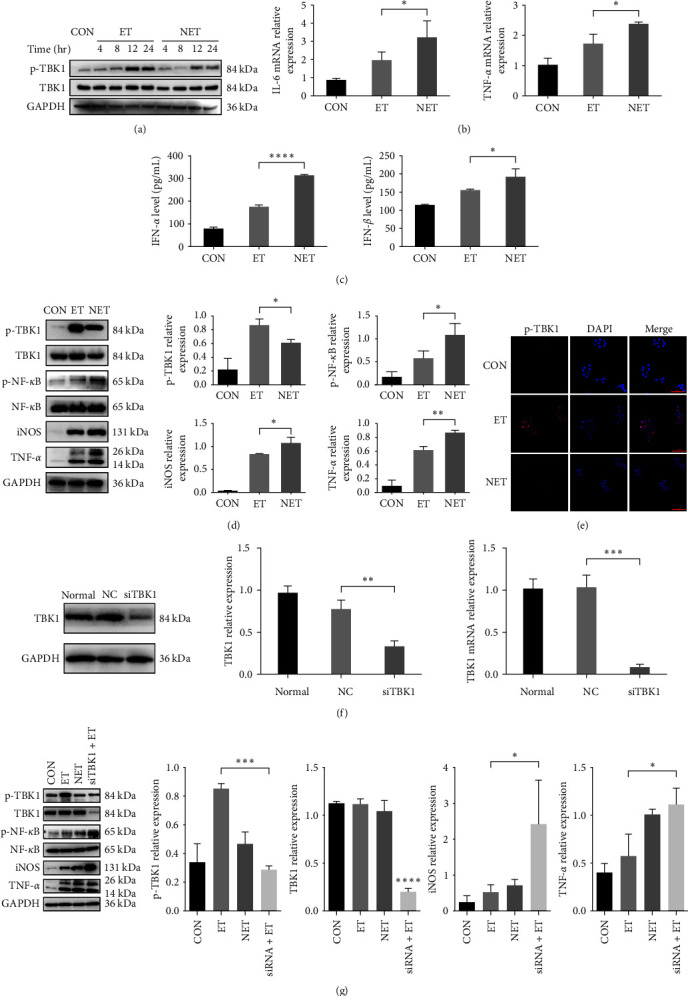
Effect of TBK1 on endotoxin tolerance in KCs in vitro: (a) KCs were randomly divided into three groups. Western blotting was used to compare the levels of p-TBK1 and TBK1 in KCs; (b) qPCR analysis of IL-6 and TNF-*α* in KCs; (c) IFN-*α* and IFN-*β* in KCs culture supernatant were evaluated by ELISA; (d) Western blotting analysis of p-TBK1, TBK1, p-NF-*κ*B, NF-*κ*B, iNOS, and TNF-*α* expression. Densitometric analysis of the Western blotting data; (e) laser scanning confocal microscopy (LSCM) (400x) showed a higher TBK1 phosphorylation in the ET group; (f) siRNA inhibits TBK1 expression which successfully knocked down the protein and mRNA expression of TBK1; (g) differences in the protein and TBK1 phosphorylation level were found by Western blotting; one-way ANOVA with Dunnett's multiple comparisons was used. Data are represented as the mean ± SD of at least three independent experiments;  ^*∗*^*p* < 0.05,  ^*∗∗*^*p* < 0.01,  ^*∗∗∗*^*p* < 0.001,  ^*∗∗∗∗*^*p* < 0.0001.

TBK1 is involved in the NF-*κ*B signaling pathway. KCs were randomly divided into three groups to investigate the role of TBK1 in ET. One thousand nanogram per milliliter LPS was used to culture the ET and NET groups; specially, 10 ng/mL LPS was used to pretreat the ET group for 24 hr. WB analysis of the ET and NET groups showed that the phosphorylation of TBK1 increased, which proved that TBK1 is involved in inflammatory regulation through protein phosphorylation. However, increased TBK1 phosphorylation was found in the ET group. Total TBK1 was unaffected (Figure 1(a)).

Once the endotoxin-resistant state was established, when KCs were exposed to 1,000 ng/mL LPS for 12 hr, the level of phosphorylation of TBK1 had the greatest difference. So, the KCs were treated as previously described with LPS (1,000 ng/mL) for 12 hr. To detect whether the ET and nontolerance models were successfully established, IL-6 and TNF-*α* mRNA were analyzed. As shown in Figure 1(b), IL-6 and TNF-*α* mRNA expressions were decreased in the ET group. The expression of IFN-*α* and IFN-*β* also confirmed the lower inflammatory response in the ET group (Figure 1(c)). We also found lower NF-*κ*B activation in the ET group (Figure S1. A). As shown in Figure 1(d), ET group had lower levels of proinflammatory cytokines and higher levels of TBK1 phosphorylation. The immunocytochemistry (ICC) results were consistent with the WB results; in the ET group, TBK1 phosphorylation was higher (Figure 1(e)). Therefore, we hypothesized that TBK1 is involved in the ET of KCs.

To examine the effect of TBK1 on ET, we downregulated the expression of TBK1 using siRNA. Following siRNA knockdown of TBK1 protein and mRNA expression, RAW264.7 cells were treated to establish ET (Figure 1(f)). As previously described, the expression of TNF-*α* and iNOS was lower in the ET group and TBK1 phosphorylation was upregulated. However, this effect was reversed by the knockdown of TBK1 (Figure 1(g)). After the use of siRNA, the effect of phosphorylated TBK1 in ET was reduced, which confirmed that the reduction of endotoxin resistance-mediated inflammation in vitro was partially dependent on TBK1.

### 3.2. TBK1 Is Required for Endotoxin Tolerance In Vivo

**Figure 2:**
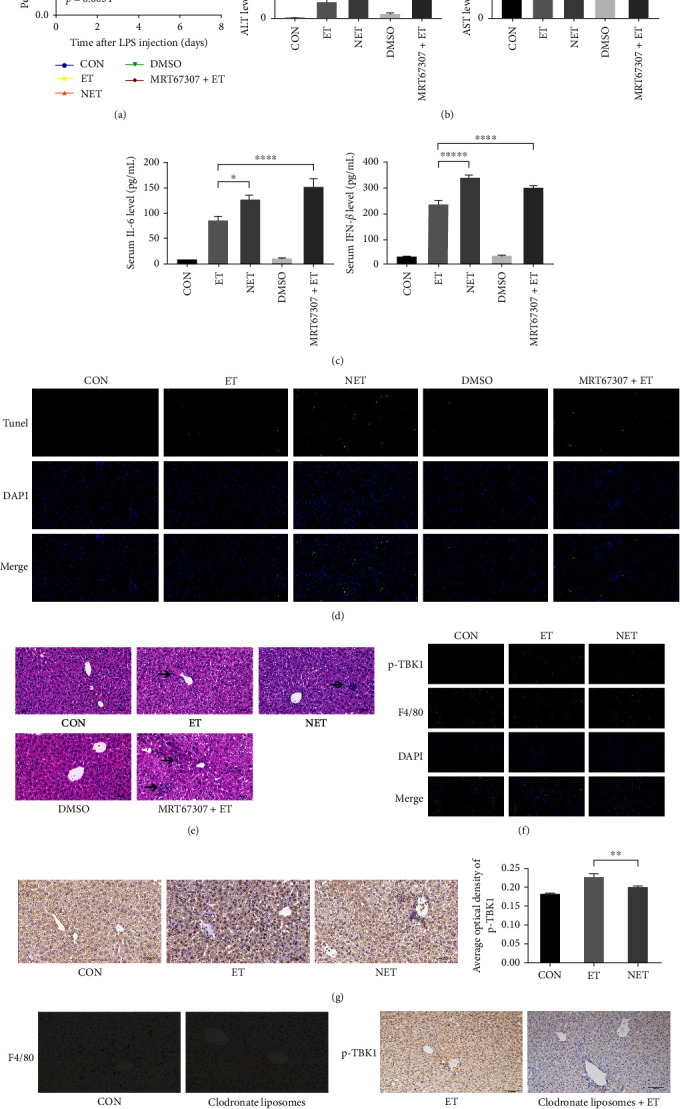
Effect of TBK1 on endotoxin tolerance in vivo: (a) ten mice in the control, endotoxin tolerance, nonendotoxin tolerance, DMSO, and TBK1-knockdown endotoxin tolerance were observed to plot survival curves. According to the log-rank test, *p* = 0.0034; (b) serum AST and ALT levels in each group of mice reflect changes in liver function; (c) the level of IL-6 and IFN-*β* in serum was evaluated by ELISA; (d) TUNEL staining was used to detect the number of apoptotic cells; (e) H&E (400x) staining of the liver showed LPS-induced liver injury; (f) immunofluorescence staining showed that the phosphorylation level of TBK1 was increased in the ET group; (g) immunohistochemical staining (400x) was used to demonstrate TBK1 phosphorylation in the liver tissues of each group; (h) clodronate liposomes were used to delete Kupffer cells in the liver. The control liposome group was 200 *μ*l control liposome and the neutral liposome group was 200 *μ*l neutral liposome; (i) phosphorylation of TBK1 in liver tissue of ET and CL + ET (Kupffer cell-depleted mice) was detected by immunohistochemical staining (400x). Statistical significance was tested using the two-tailed Student's *t*-test; data are presented as the mean ± SD of at least three independent experiments;  ^*∗*^*p* < 0.05,  ^*∗∗*^*p* < 0.01,  ^*∗∗∗*^*p* < 0.001,  ^*∗∗∗∗*^*p* < 0.0001.

For further verification, ET was established in mice as described above. We plotted survival curves and found that the survival rate of the NET group was the worst (Figure 2(a)). The serum ALT and AST levels showed less inflammatory activation and liver damage in the ET group (Figure 2(b)). Serum IL-6 and IFN-*β* levels also confirmed more severe inflammation in the NET mice (Figure 2(c)). In addition, mice in the NET group went through more hepatocyte death (Figure 2(d)). H&E staining showed more inflammatory cell infiltration (arrow) in the NET group (Figure 2(e)). The above results pointed out that in vivo, the ET model was established successfully.

As mentioned above, more phosphorylation of TBK1 was observed in the ET mouse liver (Figures 2(f) and 2(g)). To clarify the enhanced TBK1 phosphorylation observed in KCs, clodronate (neutral) liposomes were used to deplete macrophages in mice. As shown in Figure 2(h), KCs of the neutral liposome group were removed. KC-depleted mice no longer overexpress phosphorylated TBK1 even after ET (Figure 2(i)). In vivo data further confirmed the relationship between TBK1 and ET and the contribution of KCs.

MRT67307, a dual inhibitor of IKK*ε* and TBK1, was used to inhibit the expression of TBK1 in mice undergoing ET. The survival rate of ET mice with decreased TBK1 expression induced by injection of 20 mg/kg MRT67307 was not improved by the effect of ET, and these mice had the highest mortality (Figure 2(a)). Administration of 20 mg/kg MRT67307 alone did not affect the survival rate and liver tissue structure (Figure S1. B). However, after being treated with MRT67307, mice in the ET state showed higher levels of ALT, AST, serum IL-6, and IFN-*β* than ET mice (Figures 2(b) and 2(c)). The MRT67307 +ET group showed more apoptotic cells than the ET group (Figure 2(d)). Mice in the MRT67307 + ET group had multiple inflammatory cell infiltrates (arrow), even hepatocyte necrosis (Figure 2(e)). Since TBK1 was knocked down by MRT67307, the inflammatory response was still severe, even when mice have already established tolerance. These results indicate that KCs and TBK1 participate in the regulation of ET. TBK1 is essential for ET in vivo.

### 3.3. TBK1 Induces Endotoxin Tolerance by Negatively Regulating p100 Processing

**Figure 3:**
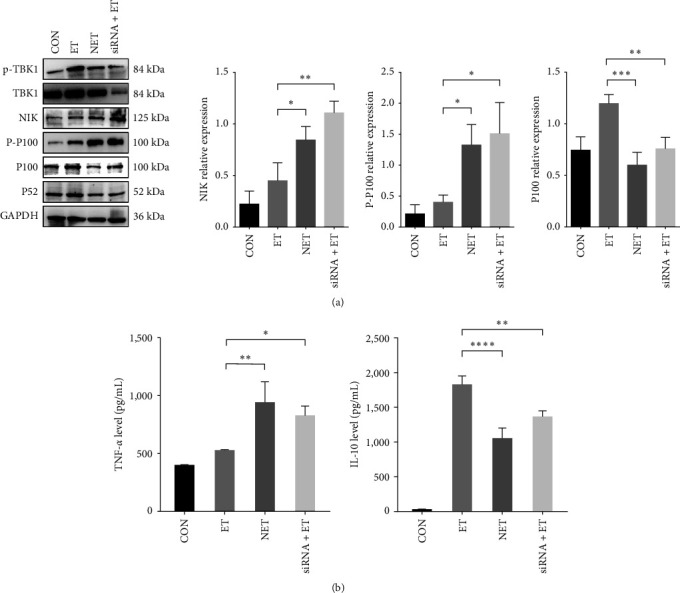
TBK1 induces endotoxin tolerance by negatively regulating p100 processing: (a) Western blotting analysis of p-TBK1, TBK1, NIK, P-P100, and NF-*κ*B/p100 protein expression. Densitometric analysis of the Western blotting data; (b) the expression of the TNF-*α* and IL-10 in the culture supernatant of RAW264.7 cells were detected by ELISA. Data are represented as the mean ± SD of at least three independent experiments; one-way ANOVA with Dunnett's multiple comparisons was used.  ^*∗*^*p* < 0.05,  ^*∗∗*^*p* < 0.01,  ^*∗∗∗*^*p* < 0.001,  ^*∗∗∗∗*^*p* < 0.0001.

Although TBK1 has been proven to be associated with ET, the regulatory mechanism of TBK1 on ET is still unclear. TBK1 is a key nonclassical IKK kinase that suppresses inflammation by phosphorylating the IKK kinase NIK and inducing its degradation [7]. NIK induces the p100 processing and nucleus translocation of the NF-*κ*B family members [19]. So, we speculate that TBK1 may induce ET through the NIK-p100 pathway. Under basal conditions, NIK is constitutively degraded by the proteasome. Consistent with previous reports [20], NIK accumulated after LPS stimulation. The NET group had higher NIK accumulation than the ET group. In the ET model, the expression of NIK decreased and then increased after siRNA inhibited the expression of TBK1 (Figure 3(a)). Figure 3(a) confirmed that TBK1 could negatively regulate the NIK expression.

NF-*κ*B/p100 was critically involved in suppressing LPS-induced inflammatory response in ET. NF-*κ*B/p100 limits p65/RelA dimer activation and nuclear translocation [2]. Consistent with assumptions, the ET group has higher expression of NF-*κ*B/p100 as a result of lower p100 processing (Figure 3(a)). When TBK1 was inhibited in the siRNA + ET group, the expression of NIK was no longer inhibited by TBK1, which caused the processing of p100. As shown in Figure 3(a), the siRNA + ET group has lower expression of NF-*κ*B/p100. TBK1 decreased in the NET group, which results in the higher expression of TNF-*α* and lower IL-10 expression (Figure 3(b)). Therefore, TBK1 may negatively regulate p100 processing by reducing the expression of NIK to induce ET. Increased p100 protein caused by high expression of TBK1 in the ET group may account for ET. Together, these data suggest that TBK1 may participate in ET by negatively regulating p100 processing.

### 3.4. The Transcription Factor c-Myb Can Negatively Regulate DTX4 to Induce the K48-Linked Polyubiquitination Profile of TBK1

**Figure 4:**
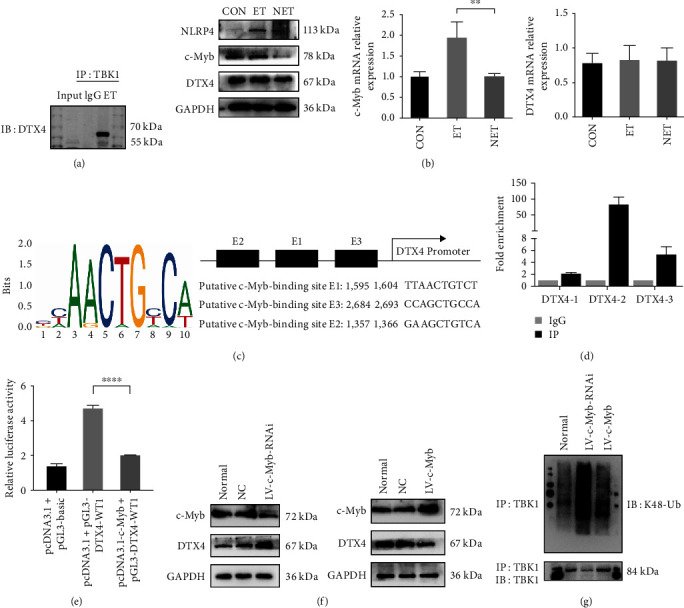
The transcription factor c-Myb can negatively regulate DTX4 to induce the K48-linked polyubiquitination profile of TBK1: (a) the interaction between TBK1 and DTX4 in KCs was verified by immunoprecipitation (IP); (b) the protein expression of NLRP4, c-Myb, and DTX4 in KCs was detected by Western blotting. Densitometric analysis of the Western blotting data; (c) position weight matrix of transcription factor c-Myb binding site (right panel). The c-Myb binding sites and the first three binding sequences of the DTX4 promoter regions were obtained from the JASPAR website (left panel); (d) the ChIP assay was performed in HEK293T cells to verify the three binding sequences of c-Myb in the DTX4 promoter region; (e) luciferase reporter assay was performed in HEK293T cells to verify that transcription factor c-Myb could negatively regulate DTX4; (f) the regulation of c-Myb on DTX4 in RAW264.7 cells was detected by Western blotting; (g) K48-linked polyubiquitination of TBK1 in RAW264.7 cells was detected. Data are represented as the mean ± SD of at least three independent experiments; one-way ANOVA with Dunnett's multiple comparisons was used.  ^*∗∗*^*p* < 0.01,  ^*∗∗∗∗*^*p* < 0.0001.

We have confirmed that TBK1 is required for ET. This means that we can effectively regulate ET by adjusting TBK1. Among the regulation of TBK1, the regulation of posttranslational modification is the most significant, especially ubiquitination. DTX4 is a key negative regulator of ubiquitination-mediated degradation of TBK1 [15]. To verify whether DTX4 could regulate TBK1 in KCs, the binding of TBK1 to DTX4 was verified by coimmunoprecipitation. Unsurprisingly, TBK1 can interact with DTX4 when KCs underwent ET (Figure 4(a)). Besides, TBK1 can also interact with DTX4 when KCs in a steady state and exposed to high-dose LPS (Figure S1. C).

DTX4 could form a complex with NRLP4 to catalyze the K48-ub of TBK1. Interestingly, NRLP4 was highly expressed in the ET group, whereas we found no difference in DTX4 protein and mRNA expression between the ET and the NET groups (Figure 4(b)). Therefore, by regulating the transcription level of DTX4, we can quickly change the content of DTX4 to effectively regulate TBK1. The transcription factors regulating DTX4 were predicted from the TFBD3 and the JASPAR database. Based on previous reports and our research focus, we chose c-Myb as the research object. The c-Myb binding site and the first three binding sequences of the DTX4 were obtained from the JASPAR website (Figure 4(c)). Figure 4(c) also showed the position weight matrix of the transcription factor c-Myb binding site. By validate the confidence of predicted sites using ChIP assays, as shown in Figure 4(d), but not E1 (TTAACTGTCT) or E3 (CCAGCTGCCA), the most likely binding site of DTX4 in the c-Myb promoter region is E2 (GAAGCTGTCA). In addition, the luciferase reporter assay confirmed that the transcription factor c-Myb decreased luciferase activity (Figure 4(e)). Collectively, these data indicate that transcription factor c-Myb can bind to the specific transcription factor binding site of DTX4, thus negatively regulating DTX4 transcription.

To confirm that the transcription factor c-Myb could negatively regulate DTX4 in ET, c-Myb was knocked down by c-Myb-RNAi lentiviruses transduction. As predicted, c-Myb protein levels were significantly reduced after lentiviral transduction. Furthermore, DTX4 showed greater expression in the LV-c-Myb-RNAi group than in the negative control group (Figure 4(f)). Meanwhile, K48-ub-TBK1 expression was slightly increased in the LV-c-Myb-RNAi group, which caused a decrease in TBK1 expression (Figure 4(g)). Overall, these data indicate that reducing c-Myb can increase DTX4 expression to reduce TBK1 expression. Correspondingly, when the c-Myb expression was increased by c-Myb-shRNA lentiviruses transduction in LV-c-Myb successfully, DTX4 expression decreased. The decrease of DTX4 led to lower expression of K48-ub-TBK1 (Figures 4(f) and 4(g)), which means that increasing c-Myb can decrease DTX4 expression to increase TBK1 expression.

All of these findings indicated that c-Myb could repress DTX4 transcription, thus affecting the K48-linked polyubiquitination profile of TBK1.

### 3.5. Overexpression of c-Myb Enhances Endotoxin Tolerance

**Figure 5:**
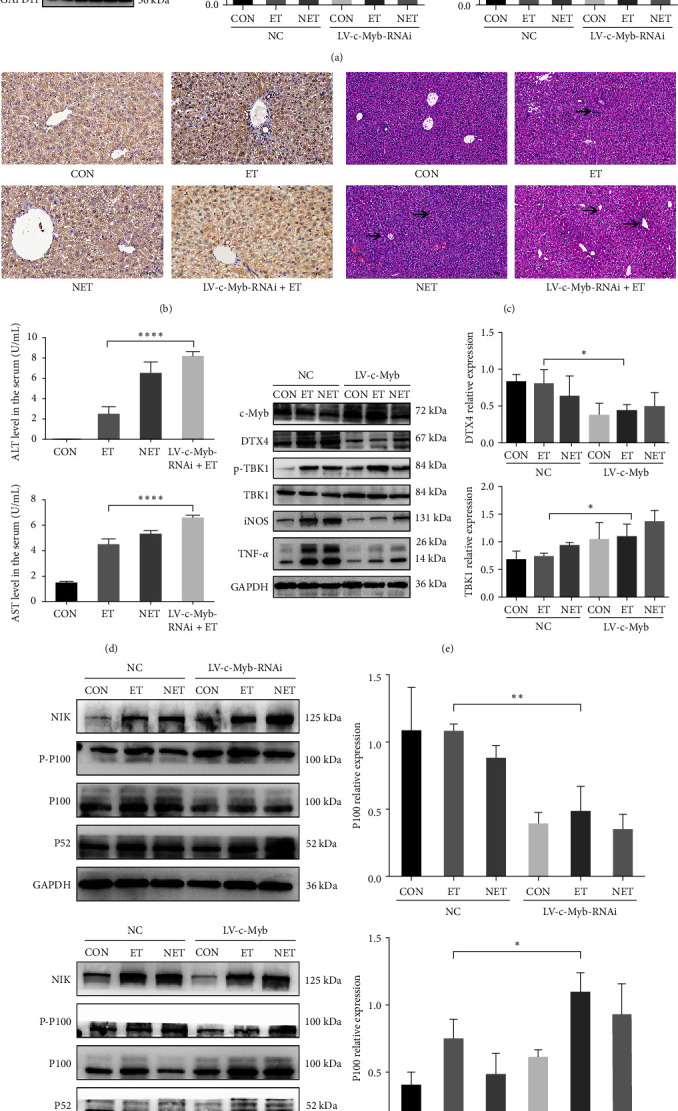
Overexpression of c-Myb enhances endotoxin tolerance: (a) lentiviral particles (LV-c-Myb-RNAi) were used to decrease c-Myb expression in RAW264.7 cells. Western blotting was used. Densitometric analysis of the Western blotting data; (b) phosphorylation of TBK1 was detected by immunohistochemical staining (400x); (c) H&E (400x) staining of liver sections in each group. Arrow indicated the infiltration of inflammatory cells; (d) detection of serum ALT and AST levels in each group. One-way ANOVA with Dunnett's multiple comparisons was used; (e) lentiviral particles (LV-c-Myb) were administered to increase c-Myb expression. Western blotting was used; (f) Western blotting results show the protein expression of NIK, P-P100, p100, and p52. Densitometric analysis of the Western blotting data. The two-tailed Student's *t*-test was used to test for statistical significance; data are represented as the mean ± SD of at least three independent experiments;  ^*∗*^*p* < 0.05,  ^*∗∗*^*p* < 0.01,  ^*∗∗∗∗*^*p* < 0.0001.

As c-Myb inhibited the luciferase activity of DTX4, we sought to examine the role of c-Myb on ET. As shown in Figure 4(b), in the ET group, the protein and mRNA expression of c-Myb were higher than that in the NET group, but protein and mRNA expression of DTX4 could not be influenced. To confirm the effect of c-Myb in ET, we knocked down c-Myb in RAW264.7 cells by LV-c-Myb-RNAi transduction. After the use of LV-c-Myb-RNAi, the expression of DTX4 increased, which can increase the K48-mediated ubiquitination of TBK1. As shown in Figure 5(a), the total TBK1 expression in the LV-c-Myb-RNAi+ET group was lower than that in the ET group. TNF-*α* and iNOS expression increased after decreasing TBK1 phosphorylation. These results demonstrate that decreasing c-Myb expression may reduce TBK1 expression, resulting in the inhibitory effect of TBK1 on inflammatory progression.

In vivo, LV-c-Myb-RNAi was injected via the tail vein [21]. Consistent with the results in vitro, Figure 5(b) shows that the phosphorylation of TBK1 in the LV-c-Myb-RNAi+ET group was lower than that in the ET group. H&E staining showed that when ET was induced by reducing the expression of c-Myb, the inflammation was aggravated (Figure 5(c)). The serum AST and ALT levels also indicated worsening after reduced c-Myb expression (Figure 5(d)). These dates supported the notion that reducing c-Myb expression is effective in attenuating the effects of ET.

To further verify the influence of c-Myb on ET and regulation of inflammation, c-Myb-shRNA lentiviruses were used to increase c-Myb expression. Compared to the negative group and the LV-c-Myb group, overexpression of c-Myb can successfully reduce the expression of DTX4. The decreased expression of DTX4 not only affects the total TBK1, but also increased the phosphorylation of TBK1. The increase of c-Myb decreased the iNOS and TNF-*α*, which means that overexpression of c-Myb increased the effect of TBK1 on ET and alleviated the inflammatory reaction. The increase in c-Myb expression alleviated the inflammatory response induced by LPS and enhanced ET (Figure 5(e)).

Consistent with previous results, the decrease of ET caused by reducing the expression of c-Myb is due to higher expression of NF-*κ*B/p100. NIK expression was higher in the LV-c-Myb-RNAi group than in the NC group, resulting in increased p100 phosphorylation and increased p100 processing (Figure 5(f)). When the expression of c-Myb increased, the increase of TBK1 decreased the expression of NIK, blocked p100 processing, and accumulated NF-*κ*B/p100 (Figure 5(f)). These results suggest that the regulation of c-Myb can dominate the process of p100 to regulate ET.

Together, our results demonstrate that c-Myb can negatively regulate DTX4, thereby contributing to ET.

## 4. Discussion

With the increasing number of Gram-negative bacterial infections and the widespread use of multidrug-resistant bacteria, ET, as a negative feedback mechanism caused by dysregulated inflammatory disorders, has attracted increasing attention [22]. Studies have shown that the regulation of ET is multilevel, involving receptors, signal molecules, negative regulators, and posttranscriptional changes [23]. In this study, the regulation of ET by TBK1 was demonstrated by establishing a classical ET model in KCs.

TBK1 is a nonclassical IKK kinase involved in the regulation of various inflammatory signaling pathways. TBK1 can degrade I*κ*B and promote NF-*κ*B activity through the phosphorylation of Ser172. However, TBK1 can also directly phosphorylate the classic IKK kinase to negatively regulate NF-*κ*B [24]. Therefore, TBK1 is thought to be involved in NF-*κ*B plasticity. TBK1 regulates endotoxin resistance as signal transduction of the NF-*κ*B signaling pathway. A significant increase in TBK1 phosphorylation was observed after LPS stimulation [25]. However, in this study, we pointed out that the phosphorylation level of TBK1 was higher in the ET group than in the NET group. Endotoxin resistance induction was abolished when TBK1 expression was inhibited by siRNA or inhibitors. Consistent with our previous study [26], we come to the conclusion that TBK1 may be a target signaling molecule for regulating excessive inflammation in KCs.

The mechanism of TBK1 regulating ET requires further in-depth study. Since TBK1 can negatively regulate the expression of NIK, in this study, we suggest that TBK1 dominates ET by negatively regulating p100 processing. When exposed to high-dose of LPS, NIK accumulates and promotes p100 processing. However, TBK1 can induce the phosphorylation of NIK and reduce NIK accumulation and p100 processing induced by LPS. p100 could be regarded as a fourth I*κ*B protein that sequesters latent NF-*κ*B dimers. NF-*κ*B2/p100 activation is important in the regulation of endotoxin resistance [27, 28]. Changing the activity of TBK1 can regulate p100 processing to regulate ET.

The ubiquitin-like domain (ULD) of TBK1 participates in the activity of TBK1 [29]. Among the posttranslational modifications of TBK1, ubiquitination is the most effective. DTX4, as an important negative regulator of TBK1, could form a complex with NRLP4 to induce K48-mediated ubiquitination of TBK1 [15]. The results revealed that DTX4 can physically interact with TBK1 during ET in KCs. NRLP4 expression was higher in the ET group than in the NET group, while TBK1 was not decreased by ubiquitination. DTX4 expression did not differ between groups. This study indicates that a change in the enrichment of DTX4 can regulate the effect of TBK1 on ET. Through a database screening, we obtained the nuclear transcription factor c-Myb that binds to specific sequences in DNA molecules. Deregulated c-Myb plays an important role in leukemia and malignant tumors [30]. ChIP experiments and dual-luciferase reporting system confirmed that c-Myb could transcriptionally negatively regulate the expression of DTX4.

Overexpression of c-Myb could reduce the transcription of DTX4, while silencing c-Myb could increase the level of DTX4, which indicated that c-Myb negatively regulated the transcription of DTX4. Our work also demonstrated that c-Myb silencing could increase DTX4 transcription, thus increasing TBK1 ubiquitination. Silencing c-Myb can reduce the effect of TBK1 on ET by weakening the inhibition of TBK1 on p100 processing. On the other hand, c-Myb overexpression could increase TBK1 phosphorylation to achieve ET by enhancing the effect of TBK1 on p100 accumulation. Interestingly, c-Myb expression decreased under LPS stimulation [31], and on this basis, overexpression of c-Myb might reduce TBK1 ubiquitination, providing a novel strategy for regulating severe inflammation.

## 5. Conclusion

Our findings provide insight into the role of TBK1 as a pivotal regulator of ET, which also demonstrates that transcription factor c-Myb can negatively regulate DTX4. Given the involvement of the potential regulatory factor in ET, our findings have implications for enhancing the understanding of ET.

## Figures and Tables

**Table 1:** Information of relevant antibodies.

Name	Catalog numbers	Company
c-Myb	ab226251	Abcam
DTX4	ab140289	Abcam
TBK1	ab40676	Abcam
TBK1	ab109735	Abcam
Phospho-TBK1/NAK (Ser172)	ab109272	Abcam
Phospho-TBK1/NAK (Ser172)	5,483	CST
iNOS	ab178945	Abcam
TNF-*α*	ab183218	Abcam
Phospho-NF-*κ*B p65 (Ser536)	3,033	CST
NF-*κ*B	10745-1-AP	Servicebio
NLRP4	NB100-56156	Novus Biologicals
NIK	4,994	CST
NF-*κ*B2 p100/p52	4,882	CST
Phospho-NF-*κ*B2 p100 (Ser866/870)	4,810	CST
DYKDDDDK tag	14,793	CST
Ubiquitin (linkage-specific K48)	ab140601	Abcam
GAPDH	GB12002	Servicebio
*β*-Actin	GB12001	Servicebio
IKK*α*	2,686	CST
IKK*β*	2,678	CST
Phospho-IKK*α*/*β*	2,697	CST

## Data Availability

The data supporting the findings of this study are available from the corresponding authors upon reasonable request.

## Supplementary Materials

Supplementary Materials Figure S1 (A) the expression of IKK*α*, IKK*β*, and P-IKK*α*/*β* in KCs in each group. Figure S1 (B) H&E (200x) staining showed that administration of MRT67307 alone did not affect liver tissue. Figure S1 (C) immunoprecipitation (IP) showed that TBK1 can interact with DTX4 in the CON and the NET group.
