# Supplementary figures and images for: c-Myb Dominates TBK1-Mediated Endotoxin Tolerance in Kupffer Cells by Negatively Regulating DTX4
Source: J Immunol Res. 2023 Mar 31;2023:5990156. doi: 10.1155/2023/5990156 (PMC10081914; doi:10.1155/2023/5990156)

Fig S1

A

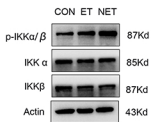

B

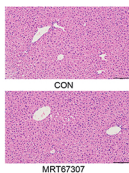

C

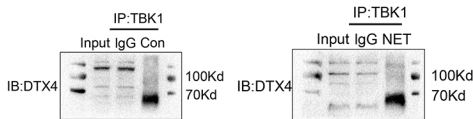

Supplement: Supplementary Materials — Figure S1 (A) the expression of IKKα, IKKβ, and P-IKKα/β in KCs in each group. Figure S1 (B) H&E (200x) staining showed that administration of MRT67307 alone did not affect liver tissue. Figure S1 (C) immunoprecipitation (IP) showed that TBK1 can interact with DTX4 in the CON and the NET group. [file 5990156.f1.pdf]
